# Improving Ebola infection prevention and control in primary healthcare facilities in Sierra Leone: a single-group pretest post-test, mixed-methods study

**DOI:** 10.1136/bmjgh-2016-000103

**Published:** 2016-12-02

**Authors:** Ruwan Ratnayake, Lara S Ho, Rashid Ansumana, Hannah Brown, Matthias Borchert, Laura Miller, Thomas Kratz, Shannon A McMahon, Foday Sahr

**Affiliations:** 1 Health Unit, International Rescue Committee, New York, New York, USA; 2 Health Unit, International Rescue Committee, Washington, District of Columbia, USA; 3 Mercy Hospital Research Laboratory, Kulanda Town, Bo, Sierra Leone; 4 Anthropology Department, Durham University, Durham, UK; 5 Institute of Tropical Medicine and International Health, Charité—Universitätsmedizin Berlin, Berlin, Germany; 6 International Rescue Committee, Freetown, Sierra Leone; 7 Information Centre for Biological Threats and Special Pathogens, Robert Koch Institute, Berlin, Germany; 8 Institute of Public Health, University of Heidelberg, Heidelberg, Germany; 9 Department of Microbiology, College of Medicine and Allied Health Sciences, University of Sierra Leone, Freetown, Sierra Leone

## Abstract

**Background:**

Accomplishing infection prevention and control (IPC) in health facilities in Sub-Saharan Africa is challenging. Owing to poor IPC, healthcare workers (HCWs) were frequently infected during Sierra Leone's Ebola epidemic. In late 2014, IPC was rapidly and nationally scaled up. We carried out workshops in sampled facilities to further improve adherence to IPC. We investigated HCW experiences and observed practice gaps, before and after the workshops.

**Methods:**

We conducted an uncontrolled, before and after, mixed-methods study in eight health facilities in Bo and Kenema Districts during December 2014 and January 2015. Quantitative methods administered to HCWs at baseline and follow-up included a survey on attitudes and self-efficacy towards IPC, and structured observations of behaviours. The intervention involved a workshop for HCWs to develop improvement plans for their facility. We analysed the changes between rounds in survey responses and behaviours. We used interviews to explore attitudes and self-efficacy throughout the study period.

**Results:**

HCWs described IPC as ‘life-saving’ and personal protective equipment (PPE) as uncomfortable for providers and frightening for patients. At baseline, self-efficacy was high (median=4/strongly agree). Responses reflecting unfavourable attitudes were low for glove use (median=1/strongly disagree, IQR, 1–2) and PPE use with ill family members (median=1, IQR, 1–2), and mixed for PPE use with ill HCWs (median=2/disagree, IQR, 1–4). Observations demonstrated consistent glove reuse and poor HCW handwashing. The maintenance of distance (RR 1.09, 95% CI 1.02 to 1.16) and patient handwashing (RR 1.19, 95% CI 1.3 to 1.25) improved to >90%.

**Conclusions:**

We found favourable attitudes towards IPC and gaps in practice. Risk perceptions of HCWs and tendencies to ration PPE where chronic supply chain issues normally lead to PPE stock-outs may affect practice. As Sierra Leone's Ebola Recovery Strategy aims to make all facilities IPC compliant, socio-behavioural improvements and a secure supply chain are essential.

**Key questions**

**What is already known about this topic?**

- A gross lack of adequate infection prevention and control (IPC) practice in health facilities was a main driver of the Ebola virus disease (EVD) epidemic in Sierra Leone.
- Given the rarity of these epidemics, it is likely that IPC strategies are not frequently documented in the scientific literature and have not undergone formal evaluation in situ.

**What are the new findings?**

- We comprehensively evaluate attitudes and self-efficacy towards IPC, and adherence to practice using the appropriate combination of qualitative, quantitative, observational and participatory approaches.
- The study was carried out during the height of the national epidemic, thereby presenting a unique opportunity to examine actual healthcare worker behaviours and attitudes under duress, and also to inform policy and practice.

**Recommendations for policy**

- Sierra Leone's National Recovery Plan for 2015–2017 has put US$33 million towards scaling up and maintaining IPC across all healthcare facilities in order to prevent a recurrence of EVD.
- The practice gaps identified provide the rationale to improve current training packages by providing insight into contextual, emotional, psychological and behavioural factors that influence adherence to IPC practice and the motivations of healthcare workers.

## Introduction

Sierra Leone was profoundly impacted by the Ebola virus disease (EVD) epidemic in West Africa, documenting 14 122 cases and 3955 deaths.^1^ Its first confirmed case in May 2014 led to the initial outbreak in the eastern districts of Kailahun and Kenema. From June to December, transmission spread to all districts and peaked at 600 confirmed cases weekly.^2^ The incidence among healthcare workers (HCWs) became 100 times that of the general population, leading to the deaths of nearly 10% of the workforce.^3^ ^4^

Poor infection prevention and control (IPC) serves as an efficient amplifier of transmission of viral haemorrhagic fevers (VHF).^5–7^ In primary healthcare facilities, also called peripheral health units (PHUs), HCWs lacked the supplies and training to apply rigorous symptom screening and IPC practices recommended for Ebola treatment units (ETU).^8^ Such deficits increased the risk of occupational and nosocomial infection for HCWs and non-EVD patients, respectively. The majority (66%) of HCW infections occurred in PHUs and hospitals.^4^ As HCWs became infected, colleagues became frightened and demoralised, and the community's trust of the health system was further eroded.^9^

By August, grossly insufficient IPC led to the infection of 43 HCWs in Kenema district, mainly in Kenema Government Hospital, which had become a de facto ETU.^3^ ^10^ To prevent EVD transmission in PHUs, the International Rescue Committee (IRC), WHO and Kenema's District Health Management Team provided IPC supplies including light personal protective equipment (PPE), and training to Kenema's PHUs near the peak of the district's outbreak in August 2014. The training covered screening, isolation, referral, hand hygiene, use of light PPE, sharps management, environmental cleaning and waste disposal.^11^ ^12^ The epidemic continued to spread rapidly and geographically. Nearly all PHUs remained open, albeit with substantially reduced staffing and services.^13^ A rapid assessment of PHUs in six districts found deficiencies in the identification and isolation of suspected cases, scarcity of supplies (PPE, chlorine, water and incinerators) and delays in referral of suspected cases to ETUs.^14^ This led the Ministry of Health and Sanitation, the IRC-led Ebola Response Consortium, UNICEF and the US Centers for Disease Control and Prevention (CDC) to train HCWs in IPC in all 1180 PHUs across 14 districts nationally, between October and December 2014.^12^ ^15^ The effort was paired with a quality assurance programme to monitor inventory, structures and practices on an ongoing basis. To learn from this experience and evaluate attitudes, experiences and the effects of an improvement workshop on behaviours, we conducted a mixed-methods study with multiple objectives. The primary objective was to generate insights into how IPC behaviours can be improved in a short time frame during an EVD outbreak. A secondary objective was to assess HCW attitudes, self-efficacy and experiences with IPC practice. Another secondary objective was to evaluate the effectiveness of participatory workshops to develop improvement plans, through the measurement of changes in adherence to IPC protocols. The primary outcome measures of effectiveness were the proportion of correct IPC behaviours within the domains of prescreening, donning, screening, doffing and consultation.

## Methods

### Study design, setting and participants

Using a participatory action framework and a mixed-methods approach, we conducted a single group, pretest post-test study (also called an uncontrolled before and after intervention study) in Bo and Kenema districts in December 2014 and January 2015.^16^ ^17^ The districts were at different phases of the epidemic. In Kenema, the epidemic had peaked, and by December, there were fewer than two cases per week. Bo's first cases were reported in July 2014, and by December, transmission dropped from 20 to 40 cases to 10 cases per week. The national IPC trainings led by the Ministry of Health and Sanitation and the Ebola Response Consortium were completed ∼1 week before the data collection for this study began in December 2014.

There were two phases of the study where data were collected: a baseline period (10–20 December 2014) and a follow-up period 3 weeks later (7–16 January 2015). The study's intervention consisted of a participatory workshop in each district immediately following the baseline period and attended by HCWs, district health officials, community health officers (CHOs, who are main healthcare provider at the PHU level) and community representatives. At this workshop, participants reviewed baseline data on IPC practices, attitudes and risk perception, and they developed improvement plans for each PHU. At baseline and follow-up, we conducted self-administered surveys with HCWs exposed to the intervention and who were present at the PHUs to assess demographics, attitudes and self-efficacy towards IPC. Also, at baseline and follow-up, we measured HCW's adherence to IPC protocols using structured observations of patient encounters. During both periods, in-depth interviews (IDIs) were conducted to explore attitudes and self-efficacy towards IPC, and experiences with IPC (without attempts to compare periods). This included vignettes where HCWs were asked how they would act in three situations related to IPC in their professional and personal lives.

**Figure 1:**
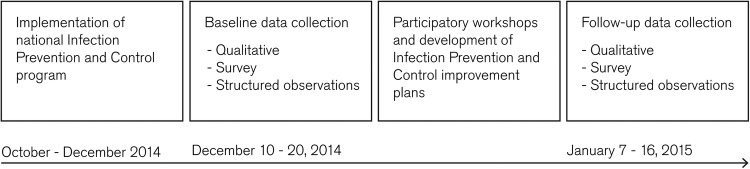
Timeline of the methods.

We used stratified random sampling to select PHUs from a sampling frame of 121 PHUs in Kenema district and of 110 PHUs in Bo district. We stratified by urban/rural setting and any/no suspected cases at the PHU level, to maximise variation. One facility was randomly chosen from each stratum in each district resulting in a total of eight participating PHUs. At least four HCWs across a range of roles were included in the IDIs at each facility as most facilities had no more than four staff. This formed the purposive sample for the survey. Sample sizes for the observations were not calculated a priori due to the fact that observers could be present in PHUs for a limited time period and therefore could capture a limited number of observations. A timeline of the methods is presented in figure 1.

### Data collection and measurement

Two observers and eight qualitative interviewers per district were trained for 2 and 3 days, respectively. Three co-investigators trained the interviewers and supervised data collection (LSH, RA and HB). Research tools were piloted in PHUs that were not selected for study. The survey was self-administered to the HCWs available on that day. For the structured observations, teams of two observers watched HCW–patient encounters for 5 hours on a single day at each PHU. Behaviours were recorded for each domain in the national protocol (patient screening, donning and doffing of PPE, patient consultation, isolation of patients screened positive, donning and doffing of PPE for isolation, and dead body management).^11^ Data were collected with smartphones using Magpi software (Datadyne, Washington, DC, USA). If a behaviour was clearly a hazard (ie, HCW attempts to touch the patient without gloves), observers were instructed to intervene. IDIs were conducted in Krio and Mende by one supervisor and three interviewers per district, digitally recorded and typed verbatim in Krio or Mende. They lasted for 30–60 min. The transcripts were translated from Krio and Mende to English.

### Data analysis

Data were analysed and interpreted concurrently using a convergent-parallel design to integrate findings across methods.^18^ Quantitative analysis of the survey and structured observations was conducted using Stata V.14 (StataCorp LP, College Station, Texas, USA). For the survey, responses on a four-point Likert item scale were summarised using the median and the IQR. Since HCWs were selected based on their availability, some HCWs may have changed between rounds. Since pairing was not possible, distributions of responses at baseline and at follow-up were compared using the Wilcoxon rank-sum test. For the structured observations, the proportion of correct behaviours for each task and the changes between rounds were computed. The main exposure and outcome were the time period (baseline vs follow-up) and the proportion of correct behaviours, respectively. A log-binomial model was used to estimate risk ratios (RR) for each correct behaviour at baseline and follow-up. Generalised estimating equations (GEE) with robust SEs accounted for repeated measures among HCWs and clustering within PHUs.^19^ An exchangeable working correlation structure was assumed. For all statistical tests, a significance level of p<0.05 was chosen. For the qualitative components, an initial phase of inductive coding on a selection of rich, diverse and representative transcripts was performed based in part on Grounded theory.^20^ Coding and analysis were conducted using Dedoose 5.011 (SocioCultural Research Consultants, LLC, Los Angeles, California, USA).

### Ethics

The study received ethics approval from Durham University's Institutional Review Board and the Sierra Leone Ethics and Scientific Research Committee. HCWs provided written informed consent. If any potentially hazardous behaviours were observed, observers were required to intervene immediately through a verbal notification to the HCW.

## Results

The survey was administered to 35 HCWs at baseline and 33 HCWs at follow-up in 8 PHUs (table 1). Twenty-two (63%) of the 35 HCWs were the same between rounds, based on profession, age and sex. There were no confirmed cases among HCWs in the sampled PHUs during the study period. Participants included CHOs, community health nurses (CHNs), maternal child health aides (MCHAs) and community health assistants (CHA). Half were below 40 years of age, and half were women. The majority (77%) were trained through the national IPC training, and 43% had already screened patients. In total, 54 IDIs were analysed. Three recordings were lost, but saturation had been reached before completion of the available transcripts. All field notes were reviewed to ensure no new themes emerged.

**Table 1:** Characteristics of survey participants, baseline (N=35)

Characteristic	N (%)
Sex, male	14 (40)
Age*	
<30	8 (23)
30–39	11 (31)
40–49	11 (31)
50+	3 (8)
Profession*	
CHN	11 (31)
MCHA	9 (26)
CHA	4 (11)
CHO	3 (9)
Community health worker	1 (3)
Endemic disease control unit assistant	1 (3)
Laboratory technician	1 (3)
Other	4 (11)
Workplace	
Community health post	17 (49)
Community health centre	16 (46)
Maternal and child health post	2 (6)
District	
Bo	16 (46)
Kenema	19 (54)
Trained in national IPC programme*	27 (77)
Screened patients in past 6 months	15 (43)

*Missing data for n=2 (age), n=1 (profession) and n=4 (training).

CHA, community health assistant; CHN, community health nurse; CHO, community health officer; IPC, infection prevention and control; MCHA, maternal child health aide.

### Implementation of the workshop intervention

Each district conducted a daylong workshop. HCWs, health authorities and community members identified key themes in the data. They developed causal diagrams and matrices, to link IPC challenges to potential solutions, and improvement plans for each PHU that aimed to improve IPC within 3 weeks (table 2). Solutions ranged from specific and attainable (eg, obtaining PPE for safe deliveries) to broad and more distal (eg, improving the water supply). Owing to the competing priorities of the emergency response, improvement plans were not always completed within 3 weeks.

**Table 2:** Key IPC challenges and solutions outlined by workshop participants in action plans

Problem	Potential solution	Frequency, n=8 (%)
Lack plan and physical materials for screening booth	Build screening materials or booth	7 (88)
Lack plan/materials for deliveries	Procure elbow gloves, delivery aprons, etc	4 (50)
No latrines for suspect cases	Build a dedicated latrine	4 (50)
Routine care requires contact	Obtain an electronic blood pressure machine	4 (50)
Community members do not understand rationale for IPC	Increase community sensitisation on IPC and handwashing	3 (38)
Handwashing among staff and patients is poor	Reinforce handwashing through signage; increase soap supply	3 (38)
Lack a working incinerator	Build an incinerator or burning pit	3 (38)
Lack an isolation area	Build an isolation area	3 (38)
Lack fencing for facility	Put in fencing	3 (38)
Water supply is inconsistent	Increase the supply of water	3 (38)
Need to reinforce supervision, training or mentorship for IPC	Implement IPC supervision or peer mentoring	2 (25)
Lack space for women postdelivery	Obtain mattresses for postnatal care	2 (25)
Concerned PPE will run out	Ensure additional PPE is available	1 (13)
Electricity is inconsistent	Address generator problems	1 (13)
Lack safe area for PPE removal	Make space for a PPE removal area	1 (13)

HCW, healthcare worker; IPC, infection prevention and control; PPE, personal protective equipment.

### Risk perception, attitudes and self-efficacy

Survey results did not change significantly between rounds; we report the baseline results in the text and the full results in table 3. Respondents believed that they had an increased risk of infection compared to the public (median=4 (strongly agree), IQR, 3–4). There was slight disagreement with the false statement that children posed a lesser risk of transmission as adults (median=2 (disagree), IQR, 2–3). HCWs described difficulty in recognising how the risks of infection for EVD and other diseases differed. As EVD was described as an epidemic, ‘it would not last for long and that maybe after one or 2 months it will all be over and gone’ (Female state enrolled nurse, Bo). When asked if they would avoid the use of gloves to treat ‘non-Ebola’ patients and PPE to treat family members for any condition, HCWs indicated strong disagreement with these statements (median=1 (strongly disagree), IQR, 1–2).

**Table 3:** Self-efficacy, risk perception and attitudes among HCWs

	Overall			Bo		Kenema	
	Baseline 35	Follow-up 33		Baseline 16	Follow-up 16	Baseline 19	Follow-up 17
No. of respondents	Median* (IQR)	Median (IQR)	p Value†	Median (IQR)	Median (IQR)	Median (IQR)	Median (IQR)
Self-efficacy							
I can correctly identify suspected Ebola cases using the screening flow chart.	4 (3–4)	3 (3–4)	0.35	4 (3–4)	4 (3–4)	4 (3–4)	4 (3–4)
I can remove PPE after isolating a suspected Ebola case without infecting myself.	4 (3–4)	3 (3–4)	0.52	4 (3–4)	3 (3–4)	4 (3–4)	3 (3–4)
I can safely disinfect a room where a suspected Ebola case has been isolated to remove any risk of infection to myself or other.	4 (3–4)	4 (3–4)	0.25	4 (3–4)	4 (3–4)	4 (3–4)	3 (3–4)
There is enough PPE at my facility to protect us from being infected with Ebola.	4 (3–4)	3 (2–4)	0.21	3 (3–4)	3 (2–4)	4 (3–4)	4 (3–4)
Attitudes and risk perception							
I am at higher risk of becoming infected with Ebola because I work in a health facility.	4 (3–4)	4 (3–4)	0.51	4 (3–4)	4 (3–4)	4 (3–4)	4 (3–4)
I am less likely to become infected with Ebola when taking care of children than adults.	2 (2–3)	2 (1–3)	0.87	2 (2–3)	2 (2–4)	2 (1–2)	2 (1–3)
If my colleague is sick it would be cruel to use PPE when treating him/her.	2 (1–4)	1 (1–3)	0.4	2 (1–4)	1 (1–2)	2 (1–4)	2 (1–4)
I do not need to use PPE when taking care of a family member with a fever, headache, diarrhoea and nausea.	1 (1–2)	1 (1–2)	0.87	1 (1–2)	1 (1–2)	1 (1–4)	1 (1–2)
I do not need to wear gloves when I take care of non-Ebola patients.	1 (1–2)	2 (1–2)	0.29	1 (1–2)	1 (1–2)	2 (1–2)	2 (1–2)

*Responses were given on a four-point Likert item scale from strongly disagree ^1^ to strongly agree ^4^.

†Evaluated using the Wilcoxon rank-sum test.

HCW, healthcare worker; IQR, interquartile range; PPE, personal protective equipment.

HCWs described PPE as uncomfortable, hot and causing sweating and itching, yet at the same time, ‘precious, lifesaving, necessary for protecting oneself and one's family’. On balance, “it's better that you overheat but are protected than that you get fresh air and become contaminated. I choose to be hot but protected” (Female CHO, Bo). A recurrent theme was that HCWs regretted the physical distance with their patients caused by PPE. There was disagreement among HCWs regarding the statement, ‘it would be cruel to use PPE when treating a sick colleague’ (median=2 (disagree), IQR, 1–4) (table 3). However, a vignette to elicit perspectives on the management of an ill HCW suggested correct behaviours. HCWs most often reported that they would tell an infected colleague to isolate herself (‘put her in observation’, ‘don't touch her’, ‘tell her not to touch anybody’) or they would refer her to an ETU (‘call the emergency line’, ‘get that ambulance to take her away’, ‘encourage her with kind words while she is being referred’). While acknowledging that it would be an upsetting experience (‘she will feel the stigma of the Ebola, she will be shedding tears, as will we’), most insisted on isolating or using PPE to treat her: “She is my colleague and friend and when the Ebola finishes…I will apologize to her, but (for now) I will not touch her, I won't do it, before all of us die, let one die so that others can live” (Female MCHA, Kenema).

Most HCWs expressed self-efficacy in identifying cases, removing PPE, and disinfecting a room after identification of a suspected case (see table 3). HCWs described five prevailing emotions that influenced the maintenance of care: disbelief, dread, fear, sadness and determination. Fear was described with the most depth and nuance, followed by sadness. Their self-efficacy developed after a gradual acceptance of the threat and after receiving training, supplies and undergoing practice. HCWs described how their own attitude or knowledge has changed after the training saying, for instance, ‘Now I feel like I have to be careful in everything I do’ (Female CHN Bo). Several HCWs, particularly those engaged in childbirth, described discontinuing work at the outset, but resuming services with confidence once they received training and PPE stocks:

Let me say the truth, before Ebola, we were working hard but we were careless in terms of IPC. As for me, the only time I used to wear gloves was during delivery…the use of chlorine for hand washing was not common…We had no idea about the use of wearing of goggles, facemasks, PPE and gowns…Now with the epidemic of Ebola, hand washing is widely practiced. (Female MCHA, Kenema)

Most HCWs mentioned that for their IPC to be effective, community sensitisation was essential. PPE induced fear among patients, evoking images of burial teams and ‘memories of brothers and sisters taken by Ebola’ and ‘buried by these people’. Sensitisation by HCWs was reportedly impeded by restrictions on their movement, inaccessibility of communities, finances and a resistance from community members:

They are really been panicked to come…they will stand at the gate and start to talk to themselves in fear of the booths that we have constructed. But we are still sensitizing them to continue coming*.* (Female MCHA, Kenema)

HCWs tried to counteract patients’ fears by counselling them individually to understand the rationale behind the use of PPE:

When the patients come, they sit down. Before we start our work, we talk to them, “Now, you see me as I am, I am alright. I am going to dress in order to protect myself, and protect you. May be I am sick but you are not aware. I would be talking to you may be the spit from my mouth jumps to your face or whatsoever or your nose or your eye being that they are closer to me, if I had the disease, you will have it. Or in case I am asking you questions then your child throws up or coughs, I will be infected. So for this reason I am going to put on these dressings. Don't see me and be afraid. I am trying to protect myself and protect you so that I won't infect you and you also will not infect me. (Male MCHA, Bo)

HCWs mentioned three further threats to self-efficacy. First, HCWs doubted the differential diagnosis for suspect cases: “typhoid…malaria…Lassa have signs of Ebola” (Female CHO, Bo). Second, respondents at follow-up remained concerned about PPE shortages (median=3 (agree), IQR, 2–3). Third, HCWs emphasised that while conducting IPC, they continued to deal with a disrupted health system:

There is no toilet, no water well, no network coverage, no means of transportation… these are our problems. … And you tell a person to wash their hands at the facility, but this is not easy without water. (HCW, Bo)

### Adherence to IPC behaviours

The proportions of correct behaviours and RRs comparing the proportion of correct behaviours between baseline (90 screenings and 54 consultations) and follow-up (131 screenings and 32 consultations) are shown in table 4 (see online supplementary material annexe (Final annex_ratnayake.pdf) for results stratified by district). No suspected cases or dead bodies were observed; therefore, all observations relate to the screening of patients and subsequent consultations. During prescreenings, only one instance of HCW handwashing was observed. The proportion of HCWs asking patients to wash their hands (RR 1.45, 95% CI 1.16 to 1.8) and patients doing so on prompting from the HCW (1.49, 1.19 to 1.86) increased. Patient handwashing, with or without HCW prompting, increased though not significantly from 82% to 99% (RR 1.21, 95% CI 0.95 to 1.71). HCWs frequently mentioned patient handwashing as straining on the HCW–patient relationship:

When they come and you tell them to wash their hands, they make comments like, “What about [you], do you wash your hands every day?”…the concept that behaviour should be changed, it is not really easy, it is difficult. (Female CHO, Kenema)

**Table 4:** Proportions of correct IPC events before and after the workshop

	Baseline n=90		Follow-up n=131		RR*	
	Correct	Per cent	Correct	Per cent		95% CI
Prescreening						
Patient went directly, or HCW-directed patient, to screening area	51	57	31	24	0.53	0.37 to 0.77
Attendant washed hands	1	1	0	0	–	–
Screener asked patient to wash hands	56	62	105	80	1.45	1.16 to 1.80
Patient washed hands on direction from HCW	54	60	105	80	1.49	1.19 to 1.86
Patient washed hands directly or washed on direction from HCW	74	82	130	99	1.27	0.95 to 1.71†
Donning						
Wore rubber boots or covers	60	67	111	85	1.51	1.14 to 1.99
Wore face shield or mask	69	77	109	83	1.27	1.03 to 1.58
Completed in correct order	3	3	73	56	8.94	0.84 to 95.61
Took off /did not wear jewellery	89	99	114	87	0.83	0.72 to 0.97
Wore new gloves	17	19	40	31	2.56	1.37 to 4.79
Continued to wear gloves	63	70	87	66	0.75	0.6 to 0.94
Screening						
No other HCWs were in screening area	86	96	104	79	0.86	0.69 to 1.07†
Stood 1.5 m from patient	82	91	130	99	1.11	0.83 to 1.48†
Sat sideways to patient	21	23	75	57	2.3	1.34 to 3.95
Held digital thermometer 5–6 cm from patient	82	91	15	12	0.23	0.12 to 0.43†
Doffing						
Removed any light PPE	13	14	42	32	2.54	1.32 to 4.88
Removed gloves	9	10	29	22	4.09	1.34 to 12.49
Washed gloved or ungloved hands	10	11	25	19	2.58	1.0 to 6.66
Removed face shield or goggles	8	9	2	2	0.21	0.05 to 0.94
Completed in correct order (if removed gloves)	3	3	29	22	6.64	2.09 to 21.14
	Baseline n=54		Follow-up n=32			
	Correct	Per cent	Correct	Per cent	RR*	95% CI
Consultations						
Washed hands before treating patient	8	15	3	10	0.63	0.18 to 2.21
Washed hands after treating patient	21	39	5	16	0.91	0.5 to 1.65
Put on new gloves before treating patient	50	93	29	91	0.97	0.85 to 1.1
Did not remove gloves after treating patient	6	11	8	25	1.51	0.55 to 4.12
Stood 1.5 m from patient	35	65	29	91	1.18	0.92 to 1.51

*Risk ratio using binomial regression (family: binomial, link: log) accounting for clustering at the health facility level (GEE). Hyphens indicate where parameter was not estimable.

†Indicates that a Poisson regression (family: Poisson, link: log) was used due to the failure of the binomial model to converge.

HCW, healthcare worker; IPC, infection prevention and control.

10.1136/bmjgh-2016-000103.supp1 Supplementary data

HCWs wore boots and face masks more than 60% of the time at baseline and more than 80% at follow-up (boots, RR 1.51, 95% CI 1.14 to 1.99; face masks, RR 1.27, 95% CI 1.03 to 1.58). Donning in the correct order increased ninefold from baseline (3%) to follow-up (56%) (RR 8.94, 95% CI 0.84 to 95.61). In 20% of screenings at follow-up, additional HCWs were present in the screening area (which is not recommended; RR 0.86, 95% CI 0.69 to 1.07). Virtually all HCWs stood 1.5 m from patients, increasing from 91% to 99% at follow-up (RR 1.11, 95% CI 0.83 to 1.48). Twice as many HCWs sat sideways towards patients to avoid bodily fluids (23% vs 57%, RR 2.3, 95% CI 1.34 to 3.95). There was a marked decrease from 91% to 12% of HCWs holding thermometers at the recommended distance of 5–6 cm from patients (RR 0.23, 95% CI 0.12 to 0.43). Across rounds, the temperature check was applied without questioning for symptoms and risk factors if afebrile. In no case did a screener ask a patient about all symptoms and risk factors. HCWs described questioning as necessary to ‘determine the [epidemiological] link’ for case identification. Still, questioning patients was not viewed as particularly effective because individuals could ‘deny and hide the (link)’.

Some differences between baseline and follow-up regarding the doffing procedure were significant, including removing light PPE and gloves (light PPE, RR 2.54, 95% CI 1.32 to 4.88 and gloves, RR 4.09, 95% CI 1.34 to 12.49) and completion in correct order (RR 6.64, 95% CI 2.09 to 21.14). Doffing was compromised by the fact that a low proportion of HCWs removed PPE between screenings (14% at baseline and 32% at follow-up). Proportions of glove removal postscreening increased, but remained low (10% at baseline, 22% at follow-up). This was accompanied by a lack of handwashing of gloved or ungloved hands between screenings (11% at baseline, 19% at follow-up). HCWs expressed concern about PPE stock-outs, as well as the strain on incinerators that frequent glove and PPE disposal would cause. Among the 29 HCWs that removed gloves, all completed doffing in the correct order at follow-up. For consultations, low proportions of HCWs washed their hands before treating a patient (15% at baseline, 10% at follow-up) or after (39% at baseline, 16% at follow-up). Most HCWs put on a new pair of gloves at baseline (93%) and follow-up (91%), and a few kept the gloves on after treating the patient. Most HCWs stayed 1.5 m from patients (65% at baseline, 91% at follow-up).

## Discussion

The EVD epidemic could be considered an overwhelming emergency in a series of severe epidemics (shigellosis and cholera) and endemic diseases (Lassa fever) in Sierra Leone that have required rigorous IPC.^21–23^ In the midst of the emergency response, we studied IPC in PHUs. This provided an exceptional opportunity to directly observe and evaluate adherence to IPC, and to work with HCWs to improve practice and discuss in detail the determinants of practice. The conviction among HCWs that IPC is lifesaving overrides the strong physical discomfort and distance with patients that it causes. During workshops, HCWs focused on improving screening, maintaining physical distance and encouraging patient handwashing; changes in these domains were reflected in the improvements seen in these behaviours at follow-up. Significant improvements were not consistent across behaviours, partly due to several high baseline values (>80%). While HCWs also discussed HCW handwashing, glove changing and the questioning for symptoms and risk factors, these were poorly adhered to across rounds.

Our study had important limitations. Uncontrolled before and after study designs lack a control group, thus limiting the ability to attribute changes observed to the intervention.^17^ Since we had a prior belief that the workshop and IPC improvement intervention would be beneficial, we believed that it would be unethical to observe IPC behaviours without intervening in a control group. Owing to the need to rapidly implement the study during a crisis, sample sizes of PHUs were intentionally small. The results are generalisable only to the PHUs included in the sample. The delay between the baseline and follow-up was short, though given the rapid progression of the epidemic, a study of short-term behaviour changes was warranted. The lack of pairing of HCWs between rounds is due to data collection being based on the availability of HCWs on the day of data collection rather than an explicit goal to conduct data collection on days when HCWs could be matched at follow-up. The implication of this limitation is that we cannot be sure that the all of those at follow-up were as exposed as those in the baseline. This likely leads to an underestimation of the intervention's effect. It is notable that staffing in PHUs is limited to a small pool of HCWs, and therefore, 63% of HCWs were the same at baseline and follow-up. As well, IPC improvement plans targeted changes at the PHU level, affecting all HCWs, not just those included in the baseline. There were gaps in fully implementing and prospectively monitoring the IPC improvement plans. Instead, we investigated changes in IPC retrospectively. At least one part of the observation protocol was apparently not adequately pretested; we think that the observations of thermometer placement at follow-up are likely specious. Transmission declined by December, limiting opportunities to assess IPC for isolation and body management; the number of HCWs observed was therefore small. Finally, HCWs who were interviewed may have been more motivated to practise IPC than those who fled during the peak of the epidemic.

Nonetheless, the quantitative and qualitative results were consistent. Attitudes towards IPC were favourable, but adherence with guidelines was markedly better for some behaviours than for others. HCWs consistently wore light PPE despite reporting persistent community fears. They described their own fear in detail, relating it to the unprecedented geographic expansion of the epidemic and the common experience of losing colleagues.^9^ We interpret this fear as being a driver for some IPC protocols. It is notable that during VHF outbreaks in Uganda and Democratic Republic of Congo, HCWs cited community resistance as a major reason for not wearing PPE in health facilities.^5^ ^24^ In contrast, PPE use in this study was high, while glove changing and handwashing among HCWs, whether gloved or ungloved, were poor. This may also reflect a gap in knowledge among HCWs about how putting on or changing gloves before making contact with patients is necessary to improve patient safety.^25^ ^26^ As gloves are fomites, changing and washing should be universal. HCW practices may be governed by the rules of rationality in disrupted health systems under normal circumstances, where chronic supply chain issues lead to widespread stock-out of PPE. Another area of uncertainty was the reported hesitation to use PPE for the management of ill colleagues. When faced with a real-life situation of an ill colleague, providers’ emotions may override their knowledge of safe practices, as seen during previous VHF epidemics.^5^ ^27^ This presents an occupational risk for HCWs who are socially and emotionally challenged by their social group's tendency to not use PPE for one of their own. Overall, as transmission had abated, underlying emotions and competing priorities may foster a waning adherence to IPC.

Our findings reveal difficulties with screening protocols in PHUs. Identifying suspect cases before they enter the PHU is the foundation for IPC in the context of EVD.^8^ Across rounds, the protocol was followed incorrectly by applying the temperature check without questioning for symptoms and risk factors if afebrile. As HCWs cited the importance of establishing epidemiologic links, one explanation for their insufficient history taking may be low confidence in the protocol's effectiveness in detecting symptoms and epidemiological links due to patients’ assumed tendency to hide them. In PHUs, the majority of patients presenting for vaccination, antenatal care, and endemic diseases would not have been infected. Making the differential diagnosis of a suspect case relies heavily on the WHO case definition that specifies symptoms similar to malaria and typhoid.^28^ The lack of questioning may indicate that HCWs exercise prescreening to judge whether a patient appears ‘well’ or ‘ill’. Patients presenting for routine services in this study may have appeared well and HCWs may have given them a cursory temperature check without appropriately questioning for risk factors (in the absence of fever). This reliance on fever may be misguided; a cohort study of confirmed cases in a holding unit at Connaught Hospital in Freetown found a reduced sensitivity of the WHO case definition with 16% of confirmed cases presenting without fever.^29^

The development of IPC systems in developing countries must address several core challenges to health systems: cost, procurement, a lack of knowledge and experience with IPC, and other cultural issues.^26^ In addition, IPC protocols may vary as the evidence base for some practices is lacking.^30^ ^31^ It follows that the rapid scale-up of the Ebola IPC protocol in Sierra Leone has been a singular challenge. In the wake of the epidemic, the importance of IPC in primary care settings elsewhere in West Africa is gaining recognition through efforts to systematically address IPC in health facilities such as the Efficiency and Edification project in Burkina Faso, Senegal and Côte d'Ivoire.^32^ Notwithstanding the structural support and costs covered by Sierra Leone's national IPC programme, there are several opportunities to improve adherence via structural, social and behavioural interventions (table 5).^33^ First, the Ebola Response Consortium's longitudinal postintervention monitoring of structures, practices and supplies is necessary for identifying improvements needed and maintaining highly specialised supervision for staff and reiterating the importance of IPC.^12^ ^15^ Second, training needs to address more complex determinants of adherence, for example, the dual aims of hand hygiene and glove changing in addressing different circumstances for contact with bodily fluids of an Ebola patient for occupational and nosocomial transmission. Explaining that gloves must be clean to protect HCWs, and their patients, is most imperative. Generating positive peer pressure through participation by colleagues and senior managers can also be a driver for adherence to hand hygiene.^26^ ^34^ Using this logic, a group of HCWs’ belief in IPC and their ability to perform it may be key to achieving consistency. Third, during the foundational training, HCWs should be engaged early in discussing the care of ill colleagues and the need to implement IPC without compromise. After an initial training, supportive supervision could probe and quell any doubts and assure the exhaustive screening of apparently healthy patients.^5^ Fourth, as community fears affect self-efficacy, sensitisation on PPE use in PHUs should be integrated into community engagement.^6^ Finally, other areas that we did not address in our study relate to the improvement of the tools of IPC which may increase HCW confidence in protocols. For instance, more research is needed to assess the effectiveness of different types of light PPE for healthcare settings^31^ ^35^ ^36^ and on the use of rapid diagnostic tests for clinical screening to improve the overall predictive value of screening for EVD.^37–39^

**Table 5:** Challenges to adherence to IPC in a primary health system

Major challenge	How addressed in December 2014–January 2015	Potential additional solutions
Communities are unprepared for the systematic use of IPC and PPE in PHUs.	HCWs sensitise community members as they come to PHU.	Targeted communication campaign in community to set expectations Counselling approaches for HCWs to use in screening and consultation
HCWs may not initially believe in the high risk of infection.	Training to raise awareness of risks for HCW infection.	Integrated IPC training in preservice education curricula Reinforcement of in-service IPC training in particular for new staff Ongoing supportive supervision
Low confidence in the identification of suspect cases.	Training in screening.	Research on new diagnostic techniques (eg, rapid diagnostic tests to increase sensitivity of the case definition and the overall effectiveness of screening)
PPE causes separation in bond between HCWs and patients.	HCWs found ways to motivate patients to recognise them.	Guidance for HCW to increase communication and bonding with patients Regular meetings between HCW and health committee to discuss issues
Discomfort while using light PPE on a routine basis.	Training in PPE use.	Technical improvements to light PPE
Poor glove changing practices. Poor handwashing.	Training in PPE use. Spot checking.	Training that emphasises reasoning for appropriate use of PPE (including risks of not changing gloves) Peer systems that emphasise changing of gloves Monitoring for feelings of high self-efficacy in core behaviours among groups of HCWs
Fear of PPE stock-out hinders use.	Routine stocking of PPE.	Improved supply chain Training that emphasises reasoning for appropriate use of PPE
Mixed attitudes towards using PPE with fellow HCWs.	No specific actions known by the authors.	Training that specifies HCW treatment scenarios and addresses doubts
Implementation within a weak and fractured health system.	IPC treated as emergency response.	Improved supply chain systems Improved payment systems for human resources Improved coverage of functional water and sanitation infrastructure

HCWs, healthcare workers; IPC, infection prevention and control; PHUs, peripheral health units; PPE, personal protective equipment.

As Sierra Leone's recovery plan intends to make all PHUs compliant with national IPC protocol, understanding how behaviours can be optimised will be paramount in achieving this goal.^40^ EVD's re-emergence in Sierra Leone in January 2016 may have led to nosocomial transmission due to the patient's treatment seeking at a hospital.^41^ ^42^ This underlines that the international community must continue to develop and support IPC in West Africa, in addition to surveillance and outbreak response mechanisms, to address future epidemics.
