# Supplementary material for: Improving Ebola infection prevention and control in primary healthcare facilities in Sierra Leone: a single-group pretest post-test, mixed-methods study
Source: BMJ Glob Health. 2016 Dec 2;1(4):e000103. doi: 10.1136/bmjgh-2016-000103 (PMC5321376; doi:10.1136/bmjgh-2016-000103)
Supplement: Supplementary data [file bmjgh-2016-000103supp.pdf]

Annex 1. Proportions of correct IPC events before and after the workshop, by district

	Bo				Kenema			
	Baseline n=45		Follow-up n=58		Baseline n=45		Follow-up n=73	
**Pre-screening**	Correct	%	Correct	%	Correct	%	Correct	%
Patient went directly, or HCW directed patient, to screening area	34	76	18	31	17	38	13	18
Attendant washed hands	0	-	0	-	1	2	0	-
Screener asked patient to wash hands	29	64	54	93	27	60	51	70
Patient washed hands upon direction from HCW	26	58	54	93	28	62	51	70
Patient washed hands directly or washed upon direction from HCW	34	76	58	100	40	89	72	99
**Donning**								
Wore rubber boots or covers	22	49	52	90	38	84	60	82
Wore face shield or mask	35	78	52	89	34	76	57	78
Completed in correct order	1	2	24	41	2	4	49	67
Took off /did not wear jewelry	44	98	52	90	45	100	62	85
Wore new gloves	12	27	31	54	5	11	9	12
Continued to wear gloves	24	53	27	47	39	87	60	82
**Screening**								
No other HCWs were in screening area	43	96	34	59	43	96	70	96
Stood 1.5 meters from patient	40	89	58	100	42	93	72	99
Sat sideways to patient	13	29	32	55	8	18	43	59
Held digital thermometer 5-6 cm from patient	40	89	2	4	42	93	13	18
**Doffing**								
Removed any light PPE	12	27	27	47	1	2	15	21
Removed gloves	8	18	27	47	1	2	2	3
Washed gloved or ungloved hands	10	22	24	41	0	-	1	1
Removed face shield or goggles	8	18	0	-	0	-	2	3
Completed in correct order (if removed gloves)	3	7	27	47	0	-	2	3
	Baseline n=26		Follow-up n=14		Baseline n=28		Follow-up n=18	
**Consultations**	Correct	%	Correct	%	Correct	%	Correct	%
Washed hands before treating	4	15	1	7	4	14	2	11
Washed hands after treating patient	17	65	3	21	4	14	2	11
Put on new gloves before treating patient	23	88	13	93	27	96	16	89
Did not remove gloves after treating patient	0	-	8	57	6	21	0	-
Stood 1.5 meters from patient	13	50	14	100	22	79	15	83
